# An Integrated Approach Involving Metabolomics and Transcriptomics Reveals Arsenic-Induced Toxicity in Human Renal Cells

**DOI:** 10.3390/toxics13060483

**Published:** 2025-06-08

**Authors:** Lin Rong, Xinxin Liang, Xingfang Zhang, Yajun Qiao, Guoqiang Li, Yuancan Xiao, Hongtao Bi, Lixin Wei

**Affiliations:** 1 Qinghai Provincial Key Laboratory of Tibetan Medicine Pharmacology and Safety Evaluation, Northwest Institute of Plateau Biology, Chinese Academy of Sciences, Xining 810008, China; ronglin@nwipb.cas.cn (L.R.);; 2 CAS Key Laboratory of Tibetan Medicine Research, Northwest Institute of Plateau Biology, Chinese Academy of Sciences, Xining 810001, China; 3 University of Chinese Academy of Sciences, Beijing 100049, China

**Keywords:** metabolomics, transcriptomics, arsenic, nephrotoxicity

## Abstract

Accumulating epidemiological evidence has indicated that arsenic exposure can lead to kidney injury. However, the underlying mechanisms of arsenic-induced nephrotoxicity have not been fully elucidated. In this study, the effect of sodium arsenite on the cell viability of HEK-293 cells was studied using a CCK-8 assay. Metabolomic and transcriptomic analyses were applied to identify differential metabolites (DMs) and differentially expressed genes (DEGs) in human renal cells exposed to arsenite, respectively. The results showed that the IC_50_ of arsenite on HEK-293 cells was 25 μM. A total of 621 DMs were identified in arsenic-treated cells (VIP > 1, *p* < 0.05). The results of the metabolome analysis revealed that purine metabolism was the major affected pathway, with 21 DMs enriched within this pathway. Additionally, 9831 DEGs were obtained after arsenic exposure (|log_2_FC| > 1, Padj < 0.05). The results of the transcriptome analysis showed that ECM–receptor interaction and cell adhesion molecules were the major altered KEGG pathways, with 54 and 70 enriched DEGs, respectively. Integrated metabolomics and transcriptomics analyses revealed that the predominant mechanisms underlying arsenic-induced nephrotoxicity were associated with the perturbations of lipid metabolism and purine metabolism. Overall, the present study provided comprehensive insights into the metabolic and transcriptional alterations in human renal cells in response to arsenic exposure, providing a referable scientific basis for subsequent arsenic-induced nephrotoxicity studies.

## 1. Introduction

Currently, arsenic contamination has gradually become a public health concern worldwide. Human exposure to arsenic mainly comes from both natural and anthropogenic sources. Geothermal activity, coal, volcanic activity, and geologic formations are the main natural sources [1]. The anthropogenic sources mainly include mining, metal smelting, the burning of fossil fuels, and the use of agricultural pesticides [2]. At present, people are often exposed to arsenic by drinking water, foodstuff consumption, and occupational exposure [3].

Studies have shown that arsenic exposure may lead to various health risks or pathological conditions, including an increased risk of cancer [4], metabolic disorders [5], diabetes [6], cardiovascular diseases [7], reproductive toxicity [8], and cognitive and behavioral impairment [9]. In addition, accumulating evidence has indicated that arsenic exposure is closely associated with kidney injury. Studies have shown that arsenic exposure could lead to histopathological changes in the kidney [10,11] and variations in blood biochemical indices reflecting renal function [12]. Mechanistic studies have shown that arsenic-induced nephrotoxicity was probably associated with oxidative stress [13], NLRP3 inflammasome activation [14], and ferroptosis [15]. However, there are still some underlying mechanisms that have not been elucidated.

In the present, an integrated approach of metabolomics and transcriptomics analyses has gradually become a newly emerging research method for toxicology [16]. Unlike previous works focusing on isolated pathways, combined multi-omics analysis allows for a more comprehensive explanation of toxicological mechanism. Our study is the first in-depth investigation of the molecular profiles underlying the toxic effects of sodium arsenite on HEK-293 cells by transcriptome and metabolome. In addition to single-omics analysis, our results also revealed joint metabolic pathways of differentially expressed genes (DEGs) and differential metabolites (DMs), offering both mechanistic insights and potential detoxification targets for arsenic poisoning.

## 2. Materials and Methods

### 2.1. Reagents

Sodium arsenite (99% purity) was purchased from Beijing Innochem Science & Technology Co., Ltd. (Beijing, China). MEM (ATCC modified) (product number: PM150467), fetal bovine serum (FBS, product number: 164210-50), 100× penicillin-streptomycin solution (product number: PB180120), phosphate-buffered saline (PBS, product number: PB180327), and 0.25% trypsin solution (product number: PB180225) were all purchased from Wuhan Pricella Biotechnology Co., Ltd. (Wuhan, China). Cell Counting Kit-8 (CCK-8, product number: AR1199) was purchased from Boster Biological Technology Co., Ltd. (Wuhan, China). All other chemicals and solvents used were of analytical grade.

### 2.2. Cell Culture

Human embryonic kidney 293 (HEK-293) cells were obtained from Wuhan Pricella Biotechnology Co., Ltd. (Wuhan, China). The cells were cultured in MEM (ATCC modified) supplemented with 10% (*v*/*v*) FBS, 100 U/mL penicillin, and 100 μg/mL streptomycin at 37 °C with 5% CO_2_ in a humidified incubator.

### 2.3. Cell Viability Assays

HEK-293 cells in the logarithmic growth phase were seeded into 96-well plate at a density of 5000 cells/well. The volume of liquid added to each well was 100 μL. After incubation for 24 h, the old medium was discarded, and fresh medium containing various concentrations of sodium arsenite (0, 0.5, 1, 5, 10, 50, and 100 μM) was added to the plate. After 24 h of arsenic exposure, a CCK-8 assay was performed to measure cell viability. After a 1 h incubation at 37 °C, the absorbance of each well was measured at 450 nm using a microplate reader (PerkinElmer, Inc., Waltham, MA, USA).

### 2.4. Metabolomic Assays and Data Analysis

The cells were seeded into 10 cm culture dishes at a density of 2.5 × 10^6^ cells per dish and incubated for 24 h. Then, the old medium was removed and replaced with fresh medium containing 0 or 25 μM sodium arsenite, and the cells were cultured for another 24 h. Seven biological replicates were performed for each concentration (*n* = 7). After experimental treatment, the cells were harvested, quickly frozen in liquid nitrogen, and then kept at −80 °C until subsequent metabolomic assays. An LC-30A Ultra-High Performance Liquid Chromatography (UHPLC) system (Shimadzu Corporation, Kyoto, Japan) equipped with a Waters ACQUITY Premier HSS T3 column was used to separate the metabolites extracted from the cell samples. Mass spectrometry (MS) analysis was performed with an AB TripleTOF 6600 mass spectrometer (AB SCIEX, Concord, ON, Canada). The obtained raw data were converted into mzXML format by ProteoWizard v3.0.20269 software. Peak extraction, peak alignment, and retention time correction were performed by the XCMS program. The peaks with a detection rate lower than 50% in each group of samples were discarded. The “SVR” method was used to correct the peak area.

Principal component analysis (PCA) score plots, orthogonal partial least squares discriminant analysis (OPLS-DA) score plots, and volcano plots were generated on the MetWare Cloud (https://cloud.metware.cn (accessed on 21 February 2024). Unsupervised PCA was performed by the R package stats 3.5.1. The data were subjected to unit variance scaling before unsupervised PCA. A volcano plot was generated with Pandas 0.23.4. Differential metabolites (DMs) were filtered with the criteria of VIP > 1 and *p* < 0.05. VIP values were obtained from OPLS-DA results, which contained score plots and permutation plots and were generated using the R package MetaboAnalystR 1.0.1. The data were log transformed (log2) before OPLS-DA, and 0 values in the data were also substituted. To avoid overfitting, a permutation test (200 permutations) was performed. The heatmap was plotted using the R package ComplexHeatmap v2.11.1 based on the relative contents of DMs, which were subjected to unit variance scaling before analysis. Finally, the obtained DMs were imported into MetaboAnalyst 6.0 for pathway analysis. Pathways with *p* values < 0.05 were regarded as significantly different.

### 2.5. Transcriptome Sequencing and Data Analysis

The cells were seeded into T75 culture flasks at a density of 4 × 10^6^ cells per flask and incubated for 24 h. Then, the old medium was removed and replaced with fresh medium containing 0 or 25 μM sodium arsenite, and the cells were cultured for another 24 h. Four biological replicates were performed for each concentration (*n* = 4). After experimental treatment, the cells were harvested, and RNA samples were extracted with TRIzol^®^ reagent for library construction. The paired end libraries were prepared using an ABclonal mRNA-seq Lib Prep Kit (ABclonal Technology Co., Ltd., Wuhan, China) following the manufacturer’s instructions, and sequencing was performed with an Illumina NovaSeq 6000 instrument. The data generated from the Illumina platform were used for bioinformatics analysis.

PCA of fragments per kilobase million (FPKM) expression values was performed using the R package stats 3.5.1 on the MetWare Cloud (https://cloud.metware.cn (accessed on 30 May 2025). The data were centered before unsupervised PCA. The differentially expressed genes (DEGs) were filtered by the “DESeq2” package with the criteria of |log_2_FC| > 1 and Padj < 0.05. Gene Ontology (GO) analysis and Kyoto Encyclopedia of Genes and Genomes (KEGG) enrichment analysis were performed using the “clusterProfiler” package. GO terms and KEGG pathways with FDR < 0.05 were considered significantly enriched.

### 2.6. Reverse Transcription–Quantitative PCR (RT-qPCR)

Gene expression was validated through reverse transcription–quantitative PCR (RT-qPCR). Briefly, HEK-293 cells were seeded in T25 culture flasks at a density of 2.355 × 10^6^ cells/flask and cultured for 24 h. Then, the old medium was removed, replaced with fresh medium containing 0 or 25 μM sodium arsenite, and the cells were cultured for another 24 h. After 24 h of incubation, the arsenic-exposed and control cells were washed once with PBS buffer and collected for RNA isolation using the TaKaRa MiniBEST Universal RNA Extraction Kit (Takara Biomedical Technology (Beijing) Co., Ltd., Beijing, China). Total RNA was reverse transcribed into cDNA using the PrimeScript™ RT reagent Kit (Perfect Real Time). qPCR was conducted using ChamQ Universal SYBR qPCR Master Mix (Vazyme Biotech Co., Ltd., Nanjing, China). The *GAPDH* gene was selected as an endogenous reference gene. The primer sequences of the genes are listed in Table 1. The relative gene expression was calculated using the 2^−ΔΔCt^ method [17].

### 2.7. Integrated Analysis of Metabolomics and Transcriptomics

The KEGG IDs of DMs and gene symbols of DEGs were imported into MetaboAnalyst 6.0 for joint pathway analysis. Pathways with FDR < 0.05 were regarded as significantly different.

### 2.8. Statistical Analysis

For the CCK-8 assay, data were presented as mean ± standard deviation (SD), and one-way ANOVA was applied to assess significant differences among groups using GraphPad Prism 8.3.0. *p* < 0.05 was considered significant.

## 3. Results

### 3.1. Cell Viability and Morphology of HEK-293 Cells After Incubation with Sodium Arsenite

**Figure 1:**
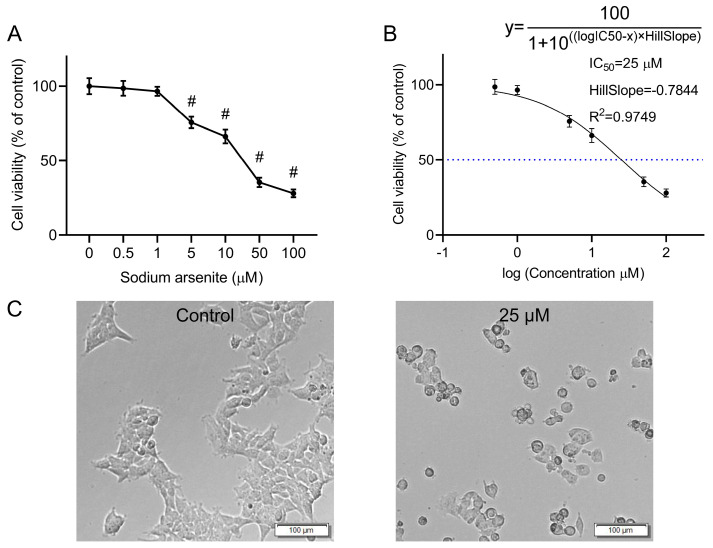
Effect of arsenic exposure on the viability and morphology of HEK-293 cells. (**A**) Cell viability of HEK-293 cells after incubation with various concentrations of sodium arsenite for 24 h. The results are shown as mean ± SD (*n* = 6). ^#^ *p* < 0.05 compared with the control group. (**B**) Nonlinear regression analysis by GraphPad Prism. The dashed line indicates the IC_50_ value. (**C**) Cell morphology of HEK-293 cells after incubation with 0 or 25 μM sodium arsenite for 24 h. Scale bar = 100 μm.

The CCK-8 assay was used to quantify the effects of various concentrations of sodium arsenite on the viability of HEK-293 cells. Sodium arsenite at low concentrations (0.5 and 1 μM) did not exert obvious effects on the cell viability, while exposure to 5, 10, 50, and 100 μM of sodium arsenite for 24 h could significantly inhibit the cell proliferation of HEK-293 cells, with cell viability of 75.62%, 66.21%, 35.41%, and 28.04%, respectively (*p* < 0.05). The above results indicated that the viability of HEK-293 cells decreased in a concentration-dependent manner after exposure to arsenic for 24 h (Figure 1A). To calculate the half maximal inhibitory concentration (IC_50_), the normalized response data were fitted to a nonlinear regression model using GraphPad Prism 8.3.0 software. The curve was generated using a four-parameter logistic model (log(inhibitor) vs. normalized response—Variable slope) (Figure 1B). The IC_50_ value was determined to be 25 μM (R^2^ = 0.9749). The HillSlope was determined to be −0.7844. In subsequent studies, 25 μM sodium arsenite was selected as the exposure concentration for further metabolomics and transcriptomics analyses.

Microscopic observation revealed obvious changes in the morphology of the arsenic-treated HEK-293 cells compared with that of the normal cells (Figure 1C). Compared with untreated cells, cells treated with 25 μΜ sodium arsenite for 24 h became wrinkled and shriveled. In addition, cytoplasmic vacuolization, which might be associated with programmed cell death [18], was also observed in some of the arsenic-treated cells.

### 3.2. Metabolomic Profiling of the HEK-293 Cells in Response to Arsenic

Untargeted metabolomic analysis was performed to analyze the alterations in the metabolic profiles after arsenic exposure. Quality control (QC) samples were prepared from a mixture of sample extracts and are usually used to analyze the reproducibility of samples under the same treatment. PCA plots showed good clustering of the QC samples, indicating great stability and reproducibility of the metabolomic assay (Figure S1).

**Figure 2:**
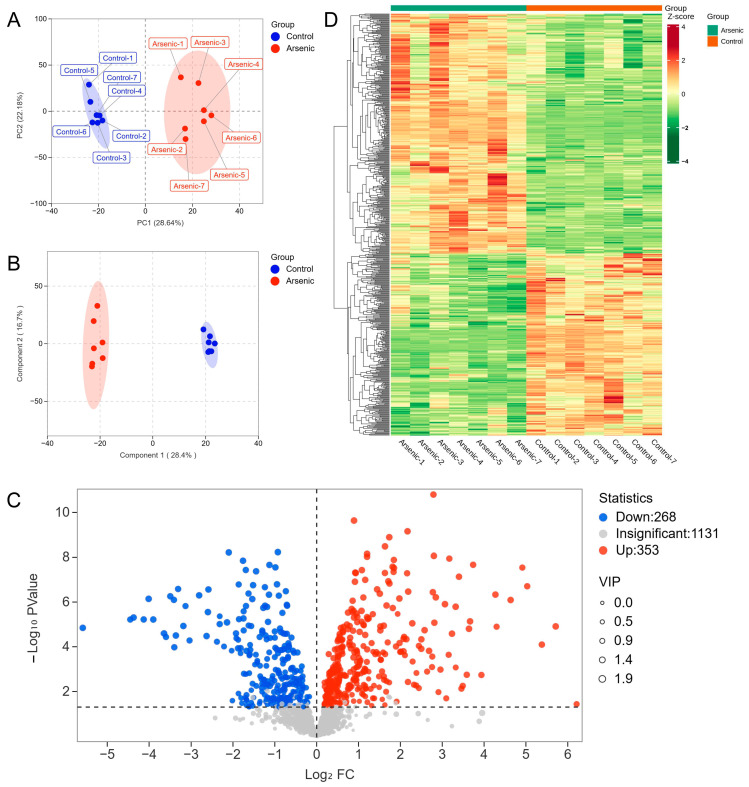
The altered metabolomic profiles of HEK-293 cells after exposure to arsenic for 24 h. (**A**) PCA score plot of metabolite data in the control and arsenic groups. (**B**) OPLS-DA score plot of metabolite data in the control and arsenic groups. (**C**) Volcano plot analysis of the DMs between the control group and arsenic group based on VIP > 1 and *p* < 0.05. Red and blue dots represent upregulated and downregulated metabolites, respectively. (**D**) Clustered heatmap analysis of DMs.

In the present study, PCA and OPLS-DA were employed to explore the metabolic changes in HEK-293 cells exposed to sodium arsenite. The PCA plot showed that the arsenic group was clearly separated from the control group, and no overlap was observed, with PC1 and PC2 explaining 28.64% and 22.18% of the variance, respectively (Figure 2A). The OPLS-DA score plot indicated that the metabolomic profiles of the arsenic group were also obviously distinguishable from those of the control group (Figure 2B). A permutation test was used to evaluate the reliability of OPLS-DA (Figure S2). As shown in the permutation validation plot, the corresponding R^2^X, R^2^Y, and Q^2^ values were 0.45, 0.996, and 0.962, respectively. As the *Y*-axis value gradually decreased, both the R^2^ and Q^2^ values gradually decreased, indicating that there was no overfitting in the original model and that the model was reliable. The combined PCA and OPLS-DA results suggested that exposure to sodium arsenite induced severe perturbation of intracellular metabolites in HEK-293 cells. A total of 621 metabolites were identified as DMs based on the standard of VIP > 1 in OPLS-DA and *p* < 0.05 in Student’s *t* test, among which 268 and 353 metabolites were downregulated and upregulated, respectively (Figure 2C). The clustering heatmap analysis showed that all the relative contents of DMs in the arsenic group were apparently different from those in the control group, also indicating that arsenic incubation could lead to apparent metabolic perturbation in HEK-293 cells (Figure 2D).

**Figure 3:**
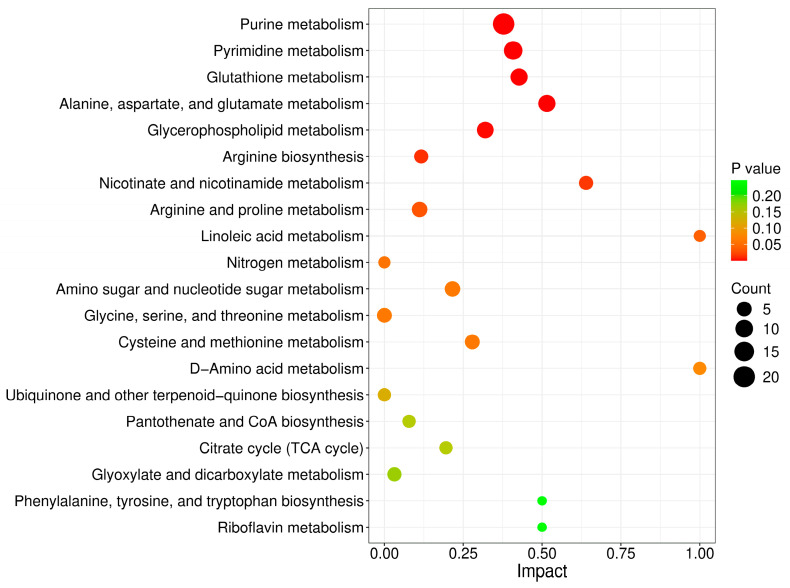
Metabolic pathways perturbed after arsenic exposure in HEK-293 cells.

To determine the metabolic pathways in which the DMs were involved, we imported these DMs into MetaboAnalyst 6.0 for pathway analysis. The *p* value represented the level of effect of sodium arsenite on the metabolic pathway. The detailed results of the top 20 pathways are summarized in Table 2. The altered metabolic pathways after arsenic exposure mainly included purine metabolism, pyrimidine metabolism, glutathione metabolism, alanine, aspartate and glutamate metabolism, and glycerophospholipid metabolism (Figure 3), among which purine metabolism was the most significantly altered pathway.

### 3.3. Transcriptomic Profiling of the HEK-293 Cells in Response to Arsenic

**Figure 4:**
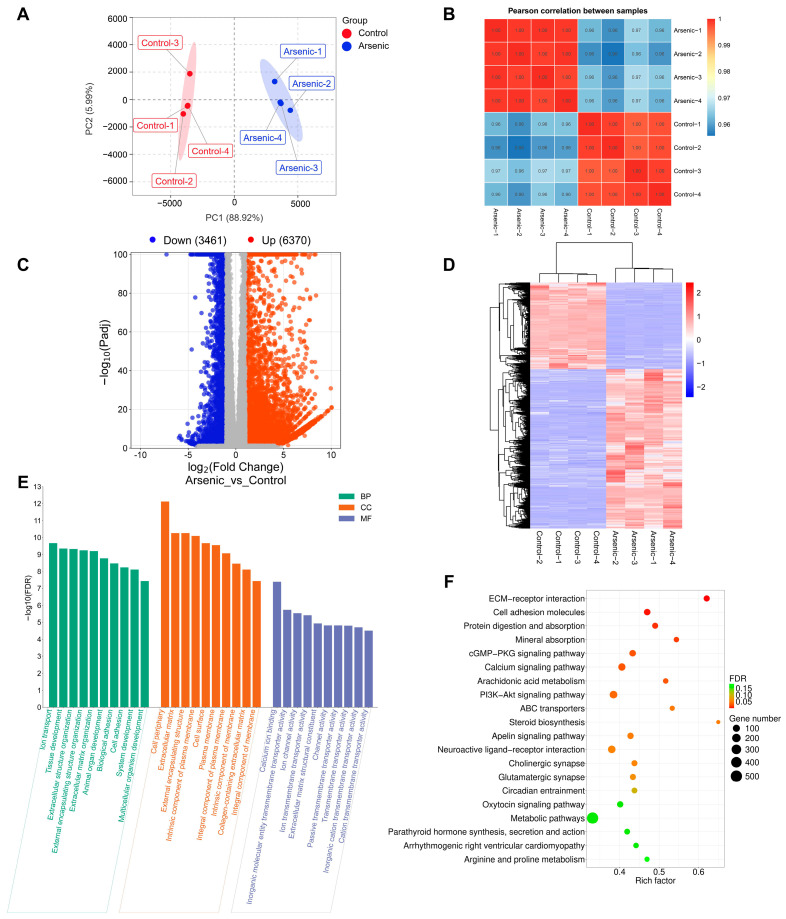
Altered transcriptome profiling of HEK-293 cells after exposure to arsenic for 24 h. (**A**) PCA score plot of the RNA-seq data. (**B**) Pearson’s correlation between samples. (**C**) Volcano plot of DEGs between the arsenic and control groups. (**D**) The cluster heatmap of DEGs upon arsenic exposure. (**E**) GO enrichment analysis of DEGs upon arsenic exposure. The top 10 BP, CC, and MF terms are shown in the figure. (**F**) KEGG pathway enrichment analysis of DEGs upon arsenic exposure. The top 20 pathways are listed in the figure.

RNA-seq analysis was employed to investigate the effects of arsenic exposure on gene expression in HEK-293 cells. The FPKM-based PCA plot showed that the arsenic group was clearly separated from the control group, which indicated that arsenic exposure had an obvious impact on the gene expression of HEK-293 cells (Figure 4A). In addition, Pearson’s correlation coefficients were used to evaluate the correlations between the different cell samples. The heatmap showed strong positive correlations among samples in the same group (Figure 4B). A total of 9831 DEGs between the arsenic and control groups were obtained based on the criteria of |log_2_FC| > 1 and Padj < 0.05. Specifically, as shown in the volcano plot (Figure 4C), 6370 upregulated DEGs and 3461 downregulated DEGs were found in HEK-293 cells exposed to 25 μM arsenic for 24 h compared with the control. The clustering heatmap analysis suggested that all the relative expression levels of DEGs in the arsenic group were apparently different from those in the control group, indicating that arsenic incubation could lead to apparent gene expression alterations in HEK-293 cells (Figure 4D). Then, the DEGs were subjected to GO and KEGG pathway enrichment analyses. As shown in Figure 4E, the GO enrichment analysis suggested that most BP terms were related to biological development, extracellular matrix organization, and biological adhesion. Most CC terms were closely related to the plasma membrane and extracellular matrix. Among the top 10 enriched MF terms, most terms were associated with molecule transmembrane transportation, such as inorganic molecular entity transmembrane transporter activity, ion channel activity, ion transmembrane transporter activity, passive transmembrane transporter activity, and transmembrane transporter activity. Among the top 20 enriched KEGG pathways (Figure 4F), ECM–receptor interaction, cell adhesion molecules, and mineral absorption were strongly associated with the above GO terms.

**Figure 5:**
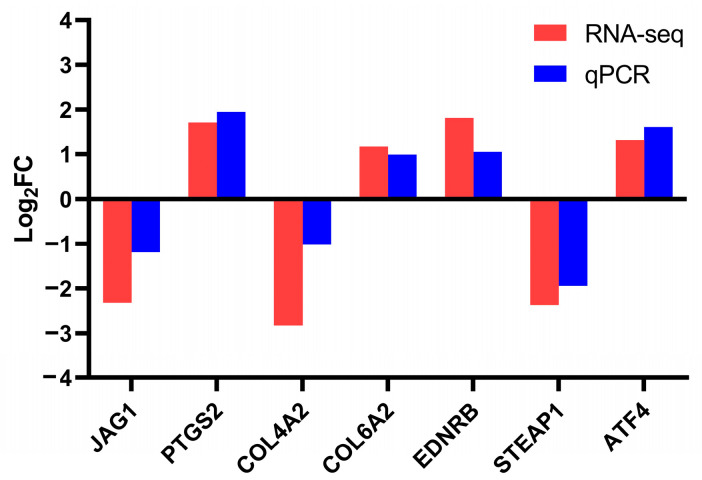
Validation of transcriptome sequencing results using RT-qPCR.

To further validate the results obtained from the transcriptomic analysis, RT-qPCR analysis was conducted on several DEGs (including *JAG1*, *PTGS2*, *COL4A2*, *COL6A2*, *EDNRB*, *STEAP1*, and *ATF4*). For RNA-seq analysis, the log_2_FC values of *JAG1*, *PTGS2*, *COL4A2*, *COL6A2*, *EDNRB*, *STEAP1*, and *ATF4* were −2.32, 1.71, −2.83, 1.17, 1.81, −2.37, and 1.32, respectively. For RT-qPCR analysis, the log_2_FC values of *JAG1*, *PTGS2*, *COL4A2*, *COL6A2*, *EDNRB*, *STEAP1*, and *ATF4* were −1.19, 1.95, −1.01, 0.99, 1.05, −1.94, and 1.61, respectively (Figure 5). The RNA-seq and RT-qPCR analyses revealed consistent expression patterns of these genes, with a Pearson’s correlation coefficient of 0.9497 (*p* = 0.0011), indicating the high reliability and accuracy of the RNA-seq data.

### 3.4. Joint Pathway Analysis of DMs and DEGs

**Figure 6:**
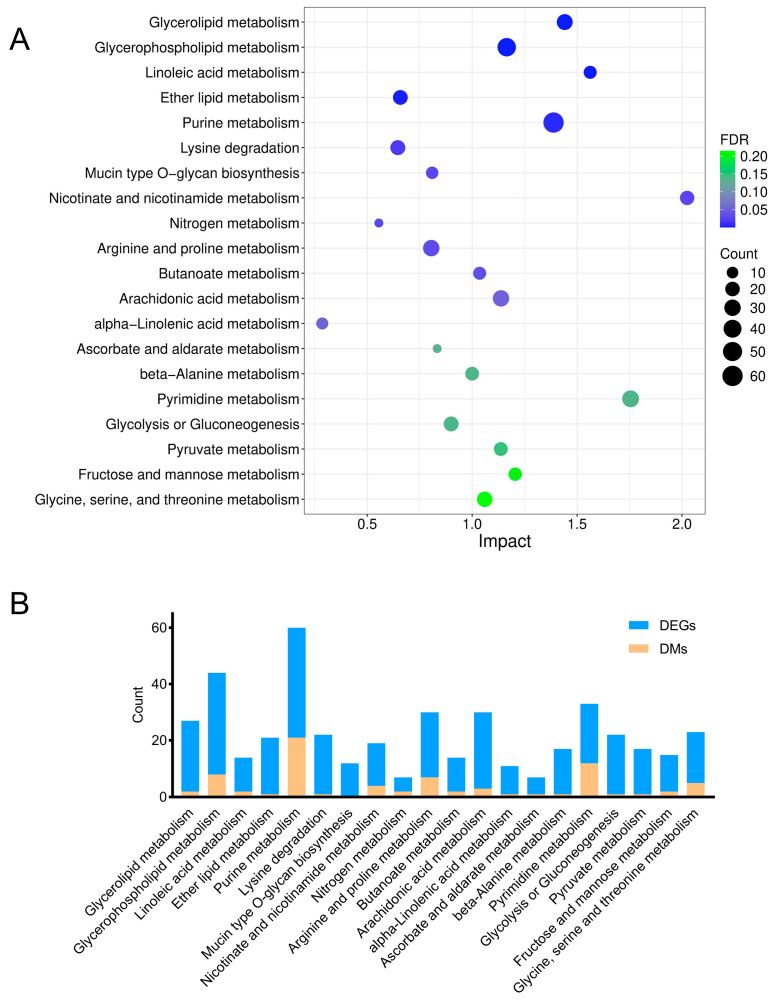
Relevant metabolic pathways perturbed after arsenic exposure based on the integrated enrichment analysis of DMs and DEGs. (**A**) Bubble plot of metabolic pathways (top 20) in which DMs and DEGs were enriched. (**B**) The number of DMs and DEGs enriched in each pathway.

To precisely elucidate the underlying mechanisms of arsenic-induced transcriptomic and metabolic disturbance, a joint pathway analysis was conducted based on the DMs and DEGs (Figure 6). The FDR represented the impact degree of sodium arsenite on the metabolic pathway. The integrated enrichment analysis indicated that arsenic exposure mainly perturbed lipid metabolism and purine metabolism in HEK-293 cells, which were further investigated in this research.

### 3.5. Lipid Metabolism Pathway Alterations

**Figure 7:**
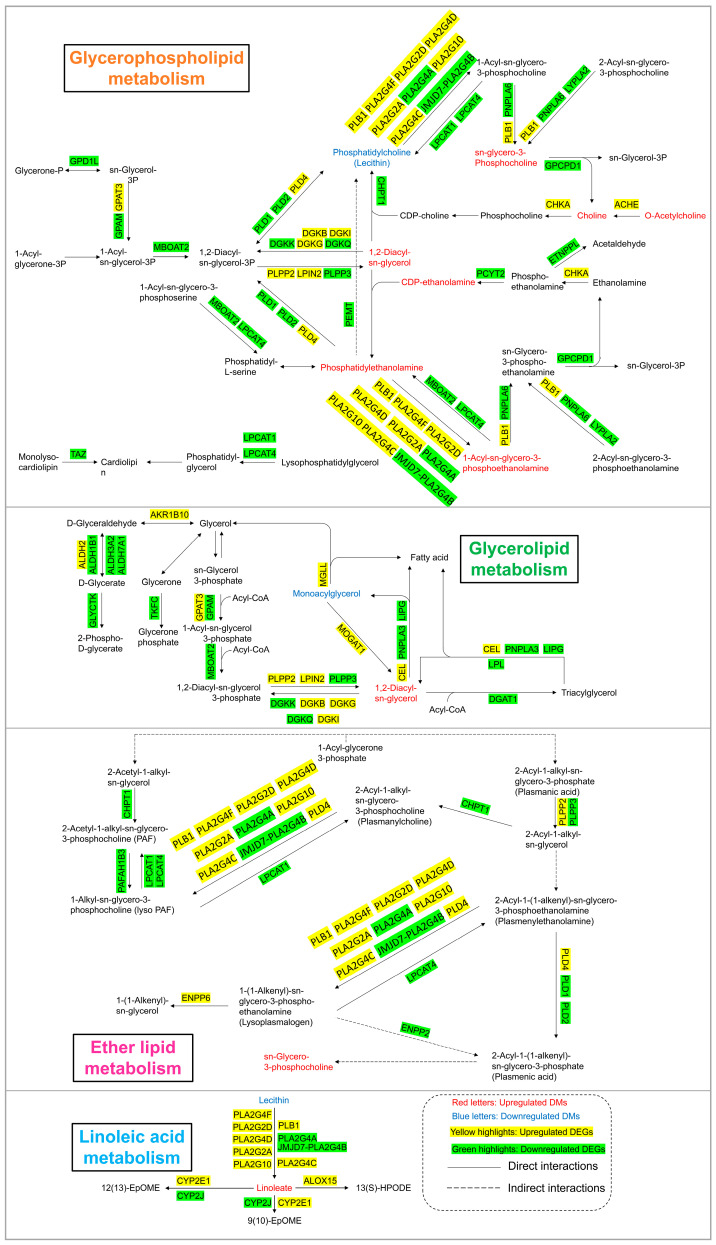
Lipid metabolism pathway alterations in HEK-293 cells after exposure to arsenic for 24 h.

As shown in Figure 6 and Figure 7, lipid metabolism, which includes pathways related to glycerophospholipid metabolism, glycerolipid metabolism, linoleic acid metabolism, and ether lipid metabolism, was obviously perturbed in HEK-293 cells after arsenic exposure. There were 8, 2, 2, and 1 DMs involved in glycerophospholipid metabolism, glycerolipid metabolism, linoleic acid metabolism, and ether lipid metabolism, respectively. Additionally, there existed 36, 25, 12, and 20 DEGs included in glycerophospholipid metabolism, glycerolipid metabolism, linoleic acid metabolism, and ether lipid metabolism, respectively.

### 3.6. Purine Metabolism Pathway Alterations

**Figure 8:**
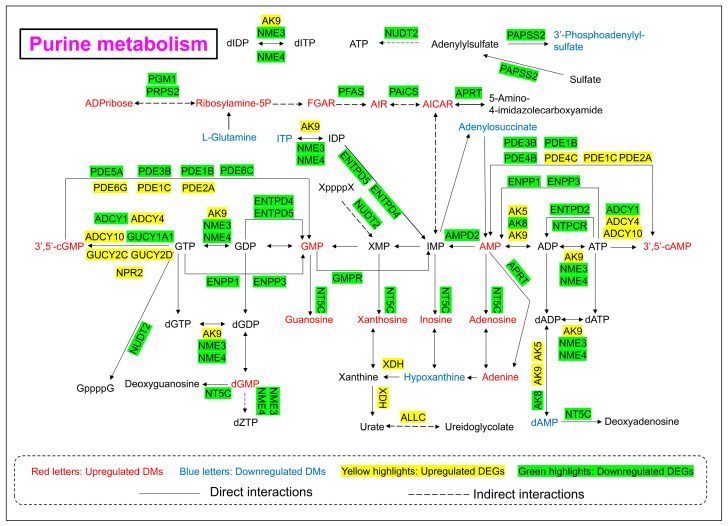
Purine metabolism pathway alterations in HEK-293 cells after exposure to arsenic for 24 h.

As shown in Figure 6 and Figure 8, the levels of 21 DMs and 39 DEGs involved in purine metabolism were significantly changed upon 24 h of arsenic exposure in HEK-293 cells. The abundances of nucleotide monophosphates (NMPs) and their deoxy counterparts (dNMPs) were obviously perturbed after arsenic exposure. The levels of adenosine monophosphate (AMP), guanosine monophosphate (GMP), deoxyguanosine monophosphate (dGMP), 3′,5′-cylic AMP, and 3′,5′-cylic GMP in the arsenic group increased to 1.17-fold, 1.72-fold, 2.80-fold, 1.95-fold, and 1.39-fold of those in the control group, respectively. However, the level of deoxyadenosine monophosphate (dAMP) in the arsenic group decreased to 0.10-fold of that in the control group. In addition, the levels of metabolites in the de novo synthesis pathway of purine nucleotides and downstream metabolites of NMPs also increased following arsenic exposure. The abundances of ADP-ribose, ribosylamine-5P, FGAR, AIR, AICAR, guanosine, xanthosine, inosine, adenosine, and adenine in the arsenic group increased to 6.92-fold, 7.03-fold, 1.73-fold, 32.69-fold, 1.83-fold, 7.77-fold, 7.52-fold, 3.05-fold, 4.22-fold, and 2.10-fold of those in the control group, respectively.

In addition, the gene expression levels of most genes corresponding to enzymes involved in purine metabolism decreased, which explained the arsenic-induced perturbation of purine metabolism to some extent. However, it is worth noting that the gene expression level of *XDH* in the arsenic group was obviously greater than that in the control group.

## 4. Discussion

A reliable in vitro cytotoxic system is essential in nephrotoxicity research. Based on previous reports [19,20], HEK-293 cells have usually been used as an effective model for investigating kidney injury and were therefore selected as an in vitro model for nephrotoxicity studies in this research. According to the results of the CCK-8 assay, the viability of HEK-293 cells decreased in a concentration-dependent manner after incubation with sodium arsenite for 24 h (Figure 1A), which was consistent with previous studies [21]. In cytotoxic experiments, the IC_50_ of a toxic substance is commonly used for toxicological studies, such as metabolomic or transcriptomic analyses [22,23]. Thus, the IC_50_ of sodium arsenite (25 μM) in HEK-293 cells was also chosen for subsequent analyses in the present study. The nephrotoxicity induced by arsenic exposure through epidemiological studies [24] or by means of laboratory model experiments [25,26] has been previously reported. However, there are still some unknown mechanisms underlying kidney injury caused by arsenic exposure. In the present study, an integrated metabolomics and transcriptomics approach was used to investigate the underlying mechanisms associated with arsenic-induced renal injury.

Metabolomics is a comprehensive analytical technique that provides a detailed overview of the small molecule metabolites present within a biological system [27]. Our investigation showed that arsenic-induced metabolic disruption in HEK-293 cells was mainly associated with changes in nucleotide metabolism, amino acid metabolism, and lipid metabolism (Figure 3). In the present, arsenic exposure has been observed to cause disturbances in amino acid metabolism and lipid metabolism [28], but there have been few studies on arsenic-induced disturbances in nucleotide metabolism. Disturbances in nucleotide metabolism, potentially attributable to elevated reactive oxygen species (ROS) levels, have been posited as a potential cause of DNA or RNA damage [29], which might deserve further research.

Transcriptomic analysis involves the comprehensive profiling of all RNA transcripts within cells or tissues and provides crucial insights into the dynamics of gene expression, regulatory networks, and cellular responses [30]. Our study revealed that KEGG pathways, such as ECM–receptor interaction and cell adhesion molecules, were obviously affected after arsenic treatment (Figure 4F). The extracellular matrix (ECM) is a well-organized dynamic three-dimensional network of biological macromolecules that offers structural support for cells and tissues. In addition to structural support, an increasing number of biological functions of the ECM, such as cell signaling, cell proliferation, differentiation, survival, and immune regulation, have been revealed [31]. Cell adhesion molecules (CAMs) promote cell-cell and ECM interactions to maintain tissue structure and normal function during homeostasis. The dysregulation of the expression and function of CAMs may lead to chronic inflammation and tissue fibrosis [32]. In this study, transcriptomic analysis revealed that ECM-related GO terms and KEGG pathways were strongly altered by arsenic exposure, suggesting that arsenic-induced nephrotoxicity is closely related to perturbations in the structure and function of the ECM. In addition, CAMs in HEK-293 cells were also obviously altered after arsenic exposure, which might also be a sign of arsenic-induced nephrotoxicity.

The integrative analysis of metabolomics and transcriptomics offers a powerful approach to link gene expression dynamics with downstream metabolic phenotypes, thus offering deeper insights into the functional mechanisms [33]. In this study, the joint pathway analysis of DMs and DEGs revealed that arsenic treatment in HEK-293 cells mainly caused the disruption of lipid metabolism and purine metabolism (Figure 6), which is discussed in detail in the following discussion.

Lipids serve as the basic components of the biological membranes of cells and the regulation of lipid metabolism, such as lipid uptake, synthesis, and hydrolysis, is important for maintaining cellular homeostasis [34]. Our study suggested that arsenic exposure induced a series of obvious changes in lipid metabolism in HEK-293 cells (Figure 7). PE and PC are considered major components of biological membranes. The altered levels of PE and PC suggested that cell membrane damage was observed in HEK-293 cells after arsenic exposure. DAG is a lipid second messenger that can interact with protein kinase C (PKC) and affect mammalian cell proliferation, survival, and motility [35]. The upregulated level of DAG suggested that arsenic exposure might perturb signaling cascade responses in HEK-293 cells.

The purine metabolism pathway has been proven to be closely linked to kidney injury. Previous studies have shown that triptolide can induce nephrotoxicity by perturbing purine and pyrimidine metabolism pathways in rats [36,37]. Similarly, our study revealed that arsenic-induced nephrotoxicity was strongly associated with the purine metabolism pathway (Figure 8). It has been reported that perturbation of nucleotide metabolism could lead to genomic instability and further carcinogenesis [38]. In this study, arsenic exposure perturbed the levels of NMPs and dNMPs, mainly AMP, GMP, dAMP, dGMP, 3′,5′-cAMP, and 3′,5′-cGMP, which might further affect the synthesis of RNA and DNA. In addition, previous studies have shown that the presence of excessive adenine is strongly associated with renal impairment. A high-adenine diet was usually used to establish an animal model of chronic renal failure (CRF) in rats [39]. In this study, the level of adenine in the arsenic group was obviously greater than that in the control group, which indicated that arsenic-induced nephrotoxicity might be associated with the accumulation of excessive adenine in renal cells. Xanthine dehydrogenase (*XDH*) is also known as xanthine oxidase (*XO*). Previous reports have shown that oxygen free radicals could be produced via xanthine oxidase in the purine metabolism pathway [40]. Thus, the high expression level of *XDH* might also be associated with arsenic-induced kidney toxicity.

## 5. Conclusions

This study investigated arsenic-induced toxicity in human renal cells using an integrated approach involving transcriptomics and metabolomics. The IC_50_ of sodium arsenite on HEK-293 cells was determined to be 25 μM, which was selected as the exposure concentration for subsequent multi-omics study. At first, the metabolomic analysis suggested that the abundances of 621 metabolites were significantly altered after arsenic exposure. The pathway analysis suggested that purine metabolism was the most significantly affected pathway in HEK-293 cells upon arsenic exposure. Afterwards, the transcriptomic analysis revealed that the expression patterns of 9831 genes were obviously altered in HEK-293 cells after arsenic exposure. The most significantly enriched GO terms were related to biological development, extracellular matrix, cell adhesion, and plasma membrane and molecule transmembrane transportation, some of which were strongly associated with significantly affected KEGG pathways such as ECM–receptor interaction, cell adhesion molecules, and mineral absorption. Finally, the joint metabolomics and transcriptomics analysis revealed that the predominant toxicological mechanisms underlying arsenic-induced nephrotoxicity were associated with the perturbation of lipid metabolism and purine metabolism. Taken together, this study provided global insights into the metabolic and transcriptional alterations in human renal cells in response to arsenic exposure, providing a referable scientific basis for subsequent arsenic nephrotoxicity studies.

## Figures and Tables

**Table 1:** Primer sequences.

Genes	Primer Sequence (5′-3′)
*JAG1*	F: CTTTGGAGCGACCTGTGTGGA
	R: ATCCCATTTGGCCCCATCTGG
*PTGS2*	F: TGCTGTTCCCACCCATGTCAA
	R: ATCCTGTCCGGGTACAATCGC
*COL4A2*	F: CATCGAATGCAATGGAGGCCG
	R: GAAGCTCTGCTCGGGAATGGT
*COL6A2*	F: ATGACGCTGTTCTCCGACCTG
	R: AAGGTCTGGGCACACGATCTG
*EDNRB*	F: AAGGAGACAGGACGGCAGGAT
	R: ACGAACACAAGGCAGGACACA
*STEAP1*	F: TGGGCATATCAACAGGTCCAACA
	R: GCCAACAGAGCCAGTATTGCC
*ATF4*	F: GAGCTGGGCAGTGAAGTGGAT
	R: TGCACTGAGGGATCATGGCAA
*GAPDH*	F: CGTCAAGGCTGAGAACGGGAA
	R: TCTCCATGGTGGTGAAGACGC

**Table 2:** Pathway analysis results based on differential metabolites.

Pathway Name	*p* Value	−log_10_(*p*)	Impact
Purine metabolism	8.9638 × 10^−9^	8.0475	0.37807
Pyrimidine metabolism	4.8149 × 10^−6^	5.3174	0.40833
Glutathione metabolism	5.5303 × 10^−5^	4.2572	0.42706
Alanine, aspartate and glutamate metabolism	5.5303 × 10^−5^	4.2572	0.51523
Glycerophospholipid metabolism	0.002264	2.6451	0.31987
Arginine biosynthesis	0.012247	1.912	0.11675
Nicotinate and nicotinamide metabolism	0.015833	1.8004	0.63948
Arginine and proline metabolism	0.032477	1.4884	0.11163
Linoleic acid metabolism	0.040641	1.391	1
Nitrogen metabolism	0.058268	1.2346	0
Amino sugar and nucleotide sugar metabolism	0.062703	1.2027	0.21591
Glycine, serine, and threonine metabolism	0.070303	1.153	0
Cysteine and methionine metabolism	0.070303	1.153	0.27873
D-Amino acid metabolism	0.077996	1.1079	1
Ubiquinone and other terpenoid–quinone biosynthesis	0.12071	0.91826	0
Pantothenate and CoA biosynthesis	0.15301	0.81529	0.07823
Citrate cycle (TCA cycle)	0.15301	0.81529	0.19528
Glyoxylate and dicarboxylate metabolism	0.17192	0.76468	0.03175
Phenylalanine, tyrosine, and tryptophan biosynthesis	0.24755	0.60633	0.5
Riboflavin metabolism	0.24755	0.60633	0.5

## Data Availability

The data presented in this study are available on request from the corresponding author.

## Supplementary Materials

The following supporting information can be downloaded at: https://www.mdpi.com/article/10.3390/toxics13060483/s1, Figure S1: PCA score plots of the control group, arsenic group, and QC in positive (POS) and negative (NEG) ion models; Figure S2: Permutation validation plots of OPLS-DA.
