# Supplementary material for: An Integrated Approach Involving Metabolomics and Transcriptomics Reveals Arsenic-Induced Toxicity in Human Renal Cells
Source: Toxics. 2025 Jun 8;13(6):483. doi: 10.3390/toxics13060483 (PMC12197441; doi:10.3390/toxics13060483)
Supplement: Supplementary file 1 [file toxics-13-00483-s001.zip › toxics-3613799-supplementary.pdf]

Supplementary Materials

An Integrated Approach Involving Metabolomics and Transcriptomics Reveals Arsenic-Induced Toxicity in Human Renal Cells

Lin Rong [1,2,3], Xinxin Liang [1,2,3], Xingfang Zhang [1,2], Yajun Qiao [1,2,3], Guoqiang Li [1,2,3], Yuancan Xiao [1,2], Hongtao Bi [1,2,*] and Lixin Wei [1,2,*]

1 Qinghai Provincial Key Laboratory of Tibetan Medicine Pharmacology and Safety Evaluation, Northwest Institute of Plateau Biology, Chinese Academy of Sciences, Xining 810008, China; ronglin@nwipb.cas.cn~(L.R.)

2 CAS Key Laboratory of Tibetan Medicine Research, Northwest Institute of Plateau Biology, Chinese Academy of Sciences, Xining 810001, China

3 University of Chinese Academy of Sciences, Beijing 100049, China

* Correspondence: bihongtao@hotmail.com (H.B.); lxwei@nwipb.cas.cn (L.W.)

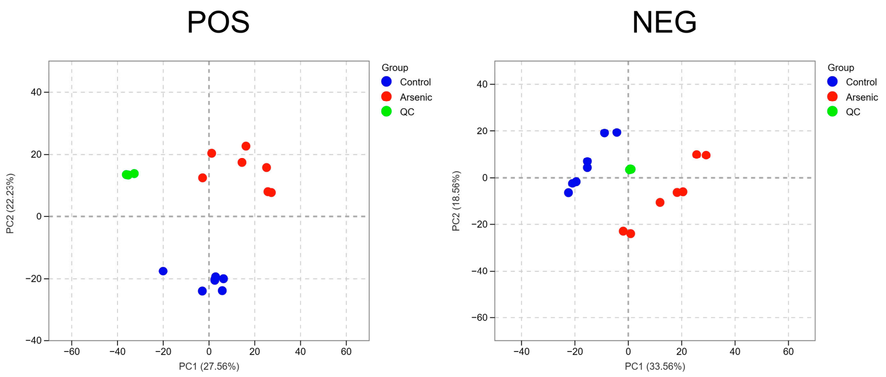

Figure S1. PCA score plots of the control group, arsenic group, and QC in positive (POS) and negative (NEG) ion models.

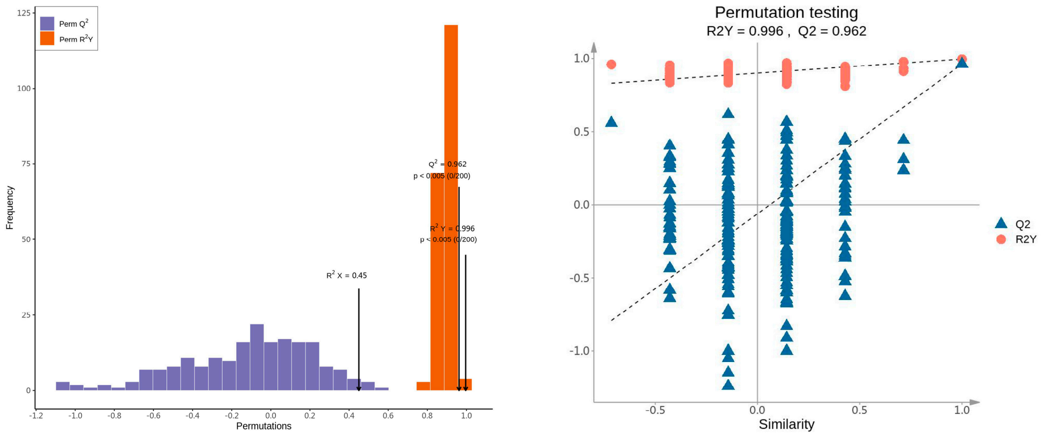

Figure S2. Permutation validation plots of OPLS-DA.
